# First‐line therapy of bevacizumab plus chemotherapy versus cetuximab plus chemotherapy for metastatic colorectal cancer patients with mucinous adenocarcinoma or mucinous component

**DOI:** 10.1002/cam4.3876

**Published:** 2021-05-03

**Authors:** Yu‐Wen Zhou, Yi‐Xiu Long, Ye Chen, Ji‐Yan Liu, Dan Pu, Jia‐Yan Huang, Feng Bi, Qiu Li, Hong‐Feng Gou, Meng Qiu

**Affiliations:** ^1^ Department of Biotherapy Cancer Center West China Hospital of Sichuan University Chengdu China; ^2^ Department of Abdominal Oncology Cancer Center West China Hospital of Sichuan University Chengdu China; ^3^ Lung Cancer Center West China Hospital Sichuan University Chengdu China; ^4^ Department of Radiology and Medical Ultrasound West China Hospital of Sichuan University Chengdu China

**Keywords:** bevacizumab, cetuximab, chemotherapy, metastatic colorectal cancer, mucinous histology

## Abstract

**Background:**

To compare the efficacy of first‐line bevacizumab plus chemotherapy with cetuximab plus chemotherapy based on the stratification of metastatic colorectal cancer (mCRC) patients with mucinous adenocarcinoma (MA) or mucinous component (MC).

**Methods:**

A retrospective study involving all mCRC patients receiving first‐line bevacizumab‐based or cetuximab‐based chemotherapy at our hospital from September 2013 to January 2020 was conducted. Overall survival (OS), progression‐free survival (PFS), and objective response rate (ORR) were compared between the cetuximab‐chemotherapy group and the bevacizumab‐chemotherapy group on the basis of the conventional pathological classification of MA or MC.

**Results:**

A total of 620 patients with mCRC were included in our study, consisting of 141 (22.7%) patients with MA/MC and 479 (77.3%) patients with non‐mucinous adenocarcinoma (NMA). In the MA/MC cohort, patients who were treated with bevacizumab‐based chemotherapy were associated with significantly better OS than those treated with cetuximab‐base chemotherapy (30.0 vs. 26.3 months, *p* = 0.002), irrespective of tumor sites. The efficacy of bevacizumab‐based chemotherapy was higher in nearly all subgroups as shown in the subgroup analysis. In the NMA cohort, median OS was better in the cetuximab plus chemotherapy group than that in the bevacizumab plus chemotherapy group (32.2 vs. 27.0 months, *p* = 0.005) for left‐side mCRC patients, whereas OS was significantly longer in the bevacizumab plus chemotherapy group for right‐side mCRC patients (26.0 vs. 20.9 months, *p* = 0.013).

**Conclusion:**

Conventional pathological classification (e.g. MA/MC) should be considered when tailoring the individualized optimal treatment for mCRC. Bevacizumab plus chemotherapy as first‐line therapy may be the optimal option for patients with MA/MC.

## 1 INTRODUCTION

Colorectal cancer (CRC) with mucinous adenocarcinoma (MA), also called mucinous colorectal adenocarcinoma (MCAC), is the second largest subtype after colorectal adenocarcinoma (CAC) and accounts for approximately 10%–15% of cases. It is defined as a tumor that is composed of ≧50% extracellular mucin on histologic examination.^1^, ^2^ MCAC is associated with specific clinicopathological characteristics such as more frequently occurring in younger individuals and in the proximal colon, has a poor differentiation, and is prone to peritoneal diffusion.^3^, ^4^, ^5^ In addition, specific molecular characteristics are also associated with mucinous differentiation. For instance, MUC2 overexpression is more common in MCAC than in CAC.^6^ Compared with CAC, MCAC is related to elevated incidence rates of CpG island methylation phenotype high, microsatellite instability (MSI)^7^ consensus molecular subtype (CMS) 1,^8^ and frequency mutations in KRAS, ERBB2, BRAF, PIK3CA, SMAD4 and GNAS genes.^9^, ^10^ In contrast, CAC with less than 50% extracellular mucin is categorized as CRC with mucinous component (MC) (CMC) and shares similar molecular and clinicopathological features with the mucinous type.^8^

The prognostic significance of patients with MA/MC remains debatable. Previous investigations indicated decreased survival in patients with MCAC,^4^, ^11^, ^12^ whereas others reported no difference in prognosis.^13^, ^14^ In the metastatic setting, MCAC was associated with an increased risk of metastasis, poor response rates to first‐line chemotherapy (resistance to irinotecan or/and oxaliplatin‐based chemotherapy),^15^, ^16^, ^17^ and decreased survival compared with CAC.^4^, ^12^, ^18^, ^19^

Monoclonal antibodies, such as bevacizumab and cetuximab, which target the vascular endothelial growth factor (VEGF) and the epithelial growth factor receptor (EGFR), respectively, in combination with 5‐fluorouracil (5‐FU)‐based chemotherapy was approved as the standard first‐line therapy for patients with metastatic CRC (mCRC).^20^, ^21^, ^22^, ^23^ Comparable^24^ or superior overall survival (OS)^21^ has been demonstrated by large clinical trials that compared first‐line treatment with cetuximab versus bevacizumab added to chemotherapy for patients mCRC. However, no studies have directly compared the efficacy of bevacizumab‐based chemotherapy with that of cetuximab‐based chemotherapy in patients with MA/MC. Previous studies have indicated that the CMS2 subtype, which potentially reinforces sensitivity to EGFR inhibition and is characterized by epithelial activation,^10^ is weakly expressed in MCAC or CMC,^25^ and MCAC is primarily constituted of the CMS1 subtype,^10^ which could achieve more survival benefit from bevacizumab than cetuximab.^26^ In this context, the purpose of this study was to compare the efficacy of first‐line bevacizumab plus chemotherapy with that of cetuximab plus chemotherapy based on the stratification of patients with MA or MC.

## 2 METHODS

### 2.1 Patients

**FIGURE 1:**
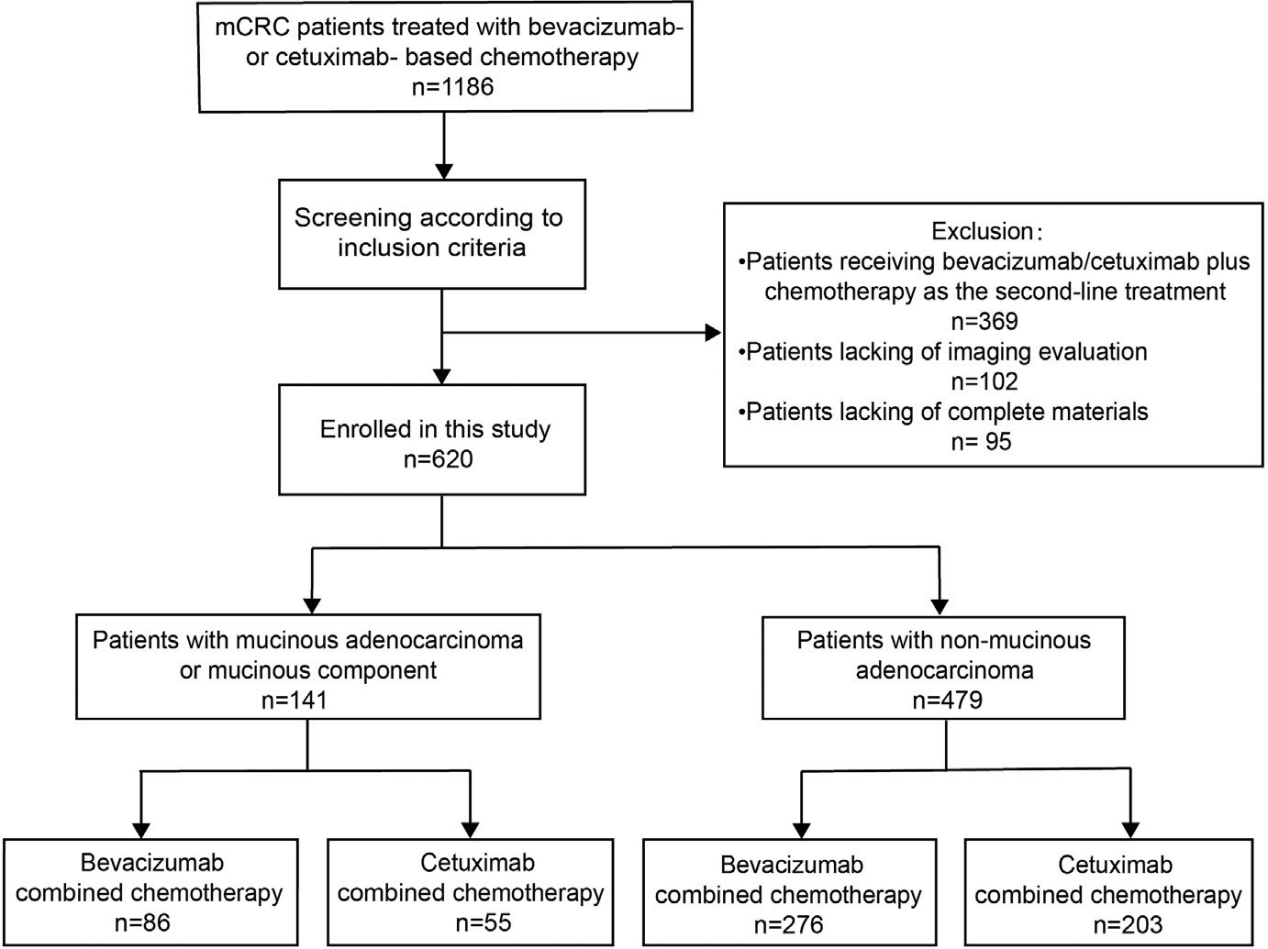
Study profile

This study was approved by the Ethics Committee of West China Hospital. Patients with mCRC received bevacizumab or cetuximab‐based chemotherapy (The dose refer to Table 1) as the first‐line treatment were reviewed at our hospital from September 2013 to January 2020. Patients were included in the analysis if they were histologically confirmed diagnosis of mCRC, either with adenocarcinoma or MA/MC; measurable lesion (based on RECIST 1.1 criteria); received bevacizumab‐ or cetuximab‐ based chemotherapy as the first‐line treatment; and at least one computerized tomography (CT)‐based therapeutic efficacy evaluation. Patients were excluded from the current study if they were lack of imaging evaluation or complete clinical materials; had underwent local treatment (surgery or radiotherapy) on measurable lesions before the first evaluation; had signet ring cells or undifferentiated components. Ultimately, 620 patients were selected for this study (Figure 1).

**TABLE 1:** The doses of bevacizumab/cetuximab combined chemotherapy

mFOLFOX6 + bevacizumab/cetuximab	Oxaliplatin 85 mg/m^2^ intravenously (i.v.) on day 1, leucovorin 400 mg/m^2^ i.v. on day 1, 5‐fluorouracil (5‐FU) 400 mg/m^2^ i.v. bolus on day 1, then total 2400 mg/m^2^ over 46–48 h i.v. continuous infusion, bevacizumab 5 mg/kg or cetuximab 500 mg/m^2^ i.v. on day 1, every 2 weeks
FOLFIRI + bevacizumab/cetuximab	Irinotecan 180 mg/m^2^ i.v. over 30–90 min on day 1, leucovorin 400 mg/m^2^ i.v. on day 1, 5‐FU 400 mg/m^2^ i.v. bolus on day 1, then total 2400 mg/m2 over 46–48 h i.v. continuous infusion, bevacizumab 5 mg/kg or cetuximab 500 mg/m^2^ i.v. on day 1, every 2 weeks
CapeOx + bevacizumab	Oxaliplatin 130 mg/m^2^ i.v over 2 h on day 1, xeloda 1000 mg/m^2^ orally twice daily on days 1–14, bevacizumab 7.5 mg/kg i.v. on day 1, every 3 weeks
FOLFOXIRI + bevacizumab	Irinotecan 165 mg/m^2^ i.v. on day 1, oxaliplatin 85 mg/m^2^ intravenously (i.v.) on day 1, leucovorin 400 mg/m^2^ i.v. on day 1, 5‐FU 400 mg/m^2^ i.v. bolus on day 1, then total 3200 mg/m^2^ over 46–48 h i.v. continuous infusion, bevacizumab 5 mg/kg i.v. on day 1, every 2 weeks

Abbreviations: CapeOx, capecitabine, oxaliplatin; FOLFIRI, folinic acid, fluorouracil, and irinotecan; FOLFOX, folinic acid, fluorouracil, and oxaliplatin; FOLFOXIRI, folinic acid, fluorouracil, irinotecan, and oxaliplatin.

### 2.2 Working methods

Based on the histology, patients were divided as MA (mucus composition >50% of tumor volume) and MC (mucinous component <50%).^1^ The classification was carried out by three pathologists with more than five years working experience from our hospitals. Pathologists were blinded to clinical results in order to avoid evaluator variability of all patients.

Clinical characteristics included age, sex, primary tumor location, pathological features, previous surgery, sites and number of metastatic diseases, chemotherapy options and RAS and BRAF status. These data were extracted and collected by three oncologists through the Hospital Information Manage System.

Response evaluation were evaluated every 2–3 months by conventional cross‐sectional imaging according to the RECIST 1.0 criteria.^27^ Objective overall response (ORR) represented a complete or partial response. Disease control rate (DCR) represented a complete, partial response or stable disease. Progression‐free survival (PFS) was calculated from the initiation of first‐line treatment to the time of tumor progression or death due to any cause. Overall survival (OS) was calculated from the initiation of first‐line treatment to the time of death from any cause. The primary survival endpoint in this study was OS. We performed follow‐up every three months. Most patients were followed up by telephone and a small number of patients were followed up with the assistance of local departments of census.

### 2.3 Statistical analysis

Categorical variables were calculated using *χ* ^2^‐test or Fisher's exact test and were performed using frequency with percentages. Survival‐based outcomes (OS and PFS) were estimated by Kaplan–Meier method. The stratified log‐rank test was applied to compare OS between two treatment groups. *p*‐value analysis was also performed using the log‐rank test. The difference of ORR between groups was analyzed by the *χ* ^2^‐test. A Cox regression model was used to estimate hazard ratio (HR) and 95% confidence intervals (95% CI). All data analysis was performed using the SPSS 25.0 software (SPSS Inc.). *p*‐value <0.05 was considered significant.

## 3 RESULTS

### 3.1 Study population and baseline characteristics

Details of the different cohort of MA/MC and non‐mucinous adenocarcinoma (NMA) population are shown in Figure 1. In short, the final study cohort is consisted of 620 patients with mCRC, including 141 (22.7%) patients with histologically confirmed diagnosis of MA/MC and 479 (77.3%) patients with NMA. The distribution characteristic between MA/MC and NMA population are shown in Table 2. Compared with NMA population, MA/MC was more common in female and young people. MA/MC was more frequently found in the proximal colon (34.8 vs. 21.7%, *p* = 0.002), with more poor differentiation (42.6 vs. 18.8%, *p* < 0.001), higher rates of lymph node (33.3 vs. 24.2%, *p* < 0.001) and peritoneal metastasis (33.3 vs. 12.9%, *p* < 0.001). Moreover, MA/MC also had a numerically high frequency of RAS mutations (43.5 vs. 39.2%, *p* = 0.414), BRAF mutation (7.5 vs. 4.8%, *p* = 0.271) and MSI (5% vs. 1.5%, *p* = 0.174). The RAS/BRAF/MMR status in the left and right colon have been detailly described in Table S2.

**TABLE 2:** The distribution characteristic between mucinous adenocarcinoma/mucinous component and non‐mucinous adenocarcinoma

Variate	Number (%)	MA/MC	NMA	*p* value
	*n* = 620 (100%)	*n* = 141 (22.7%)	*n* = 479 (77.3%)	
Age				0.308^a^
<60	355 (57.3)	86 (61.0)	269 (56.2)	
≥60	265 (42.7)	55 (39.0)	210 (43.8)	
Gender				0.928^a^
Male	367 (59.2)	83 (58.9)	284 (59.3)	
Female	253 (40.8)	58 (41.1)	195 (40.7)	
PTS				0.002^a^
Left colon	467 (75.3)	92 (65.2)	375 (78.3)	
Right colon	153 (24.7)	49 (34.8)	104 (21.7)	
Tumor differentiation				0.000^a^
Poor	150 (24.2)	60 (42.6)	90 (18.8)	
Moderate	327 (52.7)	56 (39.7)	271 (56.6)	
Well	22 (3.5)	5 (3.5)	17 (3.5)	
Unknown	121 (19.5)	20 (14.2)	101 (21.1)	
Site of metastasis				0.000^a^
Liver	416 (67.1)	77 (54.6)	339 (70.8)	
Lung	198 (31.9)	36 (25.5)	162 (33.8)	
Lymph node	166 (26.8)	47 (33.3)	119 (24.8)	
Peritoneum	110 (17.7)	47 (33.3)	63 (13.2)	
*RAS* status				0.606^a^
Wild	291 (46.9)	61 (43.3)	230 (48.0)	
Mutant	195 (31.5)	47 (33.3)	148 (31.0)	
Unknown	134 (21.6)	33 (23.4)	101 (21.1)	
*BRAF* status				0.412^a^
Wild	459 (74.0)	99 (70.2)	360 (75.2)	
Mutant	26 (4.2)	8 (5.7)	18 (3.8)	
Unknown	135 (21.8)	34 (24.1)	101 (21.1)	
*MMR* status				0.168^b^
MSS	331 (53.4)	76 (53.9)	255 (53.2)	
MSI	8 (1.3)	4 (2.8)	4 (0.8)	
Unknown	281 (45.3)	61 (43.3)	220 (45.9)	

Abbreviations: MA, mucinous adenocarcinoma; MC, mucinous component; MSI, microsatellite instability; MSS, microsatellite stabilization; NMA, non‐mucinous adenocarcinoma; PTS, Primary tumor site.

^a^ Chi square test.

^b^ Fisher's exact test.

Baseline characteristic for MA/MC and NMA population are summarized in Table 3. Of all patients with MA/MC, 86 (61.0%) cases received bevacizumab‐based chemotherapy and 55 (39.0%) cases received cetuximab‐based chemotherapy. Similarly, of all NMA patients, 276 (57.6%) cases received bevacizumab‐based chemotherapy and 203 (42.4%) cases received cetuximab‐based chemotherapy. For MA/MC patients treated with bevacizumab‐based chemotherapy, the most frequent combined regimen was FOLFIRI, while for those treated with cetuximab‐based chemotherapy, the most frequent combined regimen was mFOLFOX6. As concerning the tumor differentiation, the number of MA/MC patients with poorly differentiated in cetuximab group were significantly more than that in bevacizumab group. Other baseline features, with respect to age, sex, tumor grades, number of metastases, primary tumor resected, no statistically significant differences were observed between bevacizumab and cetuximab group either for MA/MC patients or for NMA patients. Notably, the recommendations of the National Comprehensive Cancer Network (NCCN) in 2017 were revised as anti‐EGFR therapy restricted to wild‐RAS left colorectal cancer only. As result, patients with either MA/MC or NMA were more frequently located in right‐side in bevacizumab group than those in cetuximab group.

**TABLE 3:** Baseline characteristics of eligible patients

Characteristic	MA/MC		*p* value	NMA		*p* value
	Number (%) 141 (100%)			Number (%) 479 (100%)		
	Bev‐based therapy 86 (61.0%)	Cet‐based therapy 55 (39.0%)		Bev‐based therapy 276 (57.6%)	Cet‐based therapy 203 (42.4%)	
Age	55.0 (28.0–80.0)		0.607^a^	58.0 (26.0–86.0)		0.136^a^
Age < 60	51 (59.3)	35 (63.6)		147 (53.3)	122 (60.1)	
Age ≥ 60	35 (40.7)	20 (36.4)		129 (46.7)	81 (39.9)	
Gender			0.125^a^			0.010^a^
Male	55 (64.0)	28 (50.9)		150 (54.3)	134 (66.0)	
Female	31 (36.0)	27 (49.1)		126 (45.7)	69 (34.0)	
PTS			0.003^a^			0.000^a^
Left colon	48 (55.8)	44 (80.0)		198 (71.7)	177 (87.2)	
Right colon	38 (44.2)	11 (20.0)		78 (28.3)	26 (12.8)	
Tumor differentiation			0.001^b^			0.125^b^
Poor	28 (32.6)	32 (58.2)		46 (16.7)	44 (21.7)	
Moderate	44 (51.2)	12 (21.8)		166 (60.1)	105 (51.7)	
Well	5 (5.8)	0 (0.0)		12 (4.3)	5 (2.5)	
Unknown	9 (10.5)	11 (20.0)		52 (18.8)	49 (24.1)	
T stage			0.343^b^			0.929^b^
T1	1 (1.2)	0 (0.0)		1 (0.4)	0 (0.0)	
T2	6 (7.0)	5 (9.1)		17 (6.2)	12 (5.9)	
T3	32 (37.2)	18 (32.7)		108 (39.1)	85 (41.9)	
T4	42 (48.8)	24 (43.6)		119 (43.1)	86 (42.4)	
Unknown	5 (5.8)	8 (14.5)		31 (11.2)	20 (9.9)	
N stage			0.139^b^			0.733^a^
N0	20 (23.3)	7 (12.7)		53 (19.2)	33 (16.3)	
N1	39 (45.3)	22 (40.0)		108 (39.1)	88 (43.3)	
N2	22 (25.6)	18 (32.7)		84 (30.4)	62 (30.5)	
Unknown	5 (5.8)	8 (14.6)		31 (11.3)	20 (9.9)	
NMS			0.598^a^			0.420^a^
Single	43 (50.0)	30 (54.5)		142 (51.4)	112 (55.2)	
Multiple	43 (50.0)	25 (45.5)		134 (48.6)	91 (44.8)	
Site of metastasis			0.484^a^			0.034^a^
Liver	46 (53.5)	31 (56.4)		186 (67.4)	153 (75.4)	
Lung	23 (26.7)	13 (23.6)		101 (36.6)	61 (30.0)	
Lymph node	23 (26.7)	24 (43.6)		65 (23.6)	54 (26.6)	
Peritoneum	30 (34.9)	17 (30.9)		47 (17.0)	16 (7.9)	
Other	13 (15.1)	6 (10.9)		41 (14.9)	27 (13.3)	
CCR			0.002^b^			0.080^b^
FOLFIRI	38 (44.2)	24 (43.6)		53 (19.2)	84 (41.4)	
mFOLFOX6	14 (16.3)	31 (56.4)		49 (17.7)	119 (58.6)	
CapeOX	34 (39.5)	0 (0.0)		160 (58.0)	0 (0.0)	
FOLFOXIRI	0 (0.0)	0 (0.0)		14 (5.1)	0 (0.0)	
PTR			0.706^a^			0.129^a^
Yes	60 (69.8)	40 (72.7)		179 (64.9)	145 (71.4)	
No	26 (30.2)	15 (27.3)		97 (35.1)	58 (28.6)	
BRAF status			0.093^b^			0.001^b^
Wild	55 (64.0)	44 (80.0)		195 (70.7)	165 (81.3)	
Mutation	5 (5.8)	3 (5.5)		17 (6.1)	1 (0.5)	
Unknown	26 (30.2)	8 (14.5)		64 (23.2)	37 (18.2)	
RAS status			0.000^b^			0.000^b^
Wild	13 (15.1)	48 (87.3)		64 (23.2)	166 (81.8)	
Mutation	47 (54.7)	0 (0.0)		148 (53.6)	0 (0.0)	
Unknown	26 (30.2)	7 (12.7)		64 (23.2)	37 (18.2)	

Abbreviations: Bev‐based therapy, Bevacizumab‐based therapy; CapeOx, capecitabine, oxaliplatin; CCR, Combined chemotherapy regimen; Cet‐based therapy, cetuximab‐based therapy; FOLFIRI, folinic acid, fluorouracil, and irinotecan; FOLFOX, folinic acid, fluorouracil, and oxaliplatin; FOLFOXIRI, folinic acid, fluorouracil, irinotecan, and oxaliplatin; MA, mucinous adenocarcinoma; MC, mucinous component; NMA, non‐mucinous adenocarcinoma; NMS, Number of Metastases; PTR, Primary tumor resected; PTS, Primary tumor site.

^a^ Chi square test.

^b^ Fisher's exact test.

### 3.2 Efficacy

Response and survival parameters are shown in Table 4. The median follow‐up time was 21.0 months, and the final follow‐up date was April 25th, 2020. A total of 207 patients died during the period of follow‐up, 57 in the MA/MC group and 150 in the NMA/NMC group. In all patients with MA/MC, the median OS and PFS in bevacizumab group was 30.0 months and 11.5 months significantly longer than that in cetuximab group with 26.3 months and 9.5 months, respectively (HR = 0.46, *p* = 0.002; HR = 0.65, *p* = 0.032); One patient experienced complete responses (CR) in bevacizumab group, but no CR was observed in cetuximab group (Table 5). ORR was comparable between two groups (53.5% vs. 52.7%, *p* = 0.930). By contrast, in all patients with NMA, median OS, ORR was markedly inferior in bevacizumab group compared with that in cetuximab group (27.0 vs. 29.8 months, HR = 1.56, *p* = 0.005; 52.9% vs. 67.0%, *p* = 0.002); Five patients experienced CR in cetuximab group, whereas only three patients achieved CR in bevacizumab group (Table 5). Median PFS was comparable between the two groups (10.5 vs. 10.8 months, HR = 1.10, *p* = 0.408) (Table 4).

**TABLE 4:** Effect of primary tumor location on efficacy in the colorectal cancer patients with mucinous adenocarcinoma or with mucinous component

MA/MC	Total (*n* = 141)			Left colorectal (*n* = 92)			Right colorectal (*n* = 49)		
	Bev + CT vs. Cet + CT	Crude HR (95%CI)	*p* value	Bev + CT vs. Cet + CT	Crude HR (95%CI)	*p* value	Bev + CT vs. Cet + CT	Crude HR (95%CI)	*p* value
OS	30.0 vs. 26.3	0.46 (0.27–0.79)	0.002*	31.0 vs. 28.4	0.51 (0.27–0.97)	0.029*	27.3 vs. 19.5	0.22 (0.07–0.70)	<0.001*
PFS	11.5 vs. 9.5	0.65 (0.43–1.00)	0.032*	12.4 vs. 10.5	0.65 (0.39–1.07)	0.073	10.7 vs. 7.6	0.34 (0.12–0.95)	0.002*
		OR (95%CI)			OR (95%CI)			OR (95%CI)	
ORR (%)	53.5 vs. 52.7	1.03 (0.52–2.03)	0.930	56.3 vs. 54.5	1.07 (0.47–2.44)	0.869	50.0 vs. 45.5	1.20 (0.31–4.61)	0.791

NMA	Total (*n* = 479)			Left colorectal (*n* = 375)			Right colorectal (*n* = 104)		
	Bev + CT vs. Cet + CT	Crude HR (95%CI)	*p* value	Bev + CT vs. Cet + CT	Crude HR (95%CI)	*p* value	Bev + CT vs. Cet + CT	Crude HR (95%CI)	*p* value
OS	27.0 vs. 29.8	1.56 (1.13–2.15)	0.005*	27.0 vs. 32.2	1.75 (1.16–2.64)	0.005*	26.0 vs. 20.9	0.53 (0.29–0.97)	0.013*
PFS	10.5 vs. 10.8	1.10 (0.88–1.38)	0.408	10.6 vs. 11.7	1.12 (0.86–1.45)	0.405	10.0 vs. 8.4	0.73 (0.44–1.23)	0.179
		OR (95%CI)			OR (95%CI)			OR (95%CI)	
ORR (%)	52.9 vs. 67.0	0.55 (0.38–0.81)	0.002*	54.0 vs. 69.5	0.52 (0.34–0.79)	0.002*	50.0 vs. 50.0	1.00 (0.41–2.43)	1.000

Crude HR is univariable HR.

Abbreviations: Bev, bevacizumab; Cet, cetuximab; CT, chemotherapy; MA, mucinous adenocarcinoma; MC, mucinous component; NMA, non‐mucinous adenocarcinoma; OR, odds ratio; ORR, objective overall response; OS, overall survival; PFS, progression‐free survival.

* Significant difference.

**TABLE 5:** Response to treatment

	MA/MC (*n* = 141)			*p* value	NMA (*n* = 479)			*p* value
	Total	Bev + CT (*n* = 86)	Cet + CT (*n* = 55)		Total	Bev + CT (*n* = 276)	Cet + CT (*n* = 203)	
CR	1	1	0		8	3	5	
PR	74	45	29		274	143	131	
SD	40	25	15		118	74	44	
PD	26	15	11		79	56	23	
ORR	53.2	53.5	52.7	0.930	58.9	52.9	67.0	0.002
DCR	81.6	82.6	80.0	0.702	83.5	79.7	88.7	0.009

Patients with a CR, PR, SD or PD at any time during the study.

Abbreviations: Bev, bevacizumab; Cet, cetuximab; CR, Complete response; CT, chemotherapy; MA, mucinous adenocarcinoma; MC, mucinous component; NMA, non‐mucinous adenocarcinoma; PD, Progressive disease; PR, Partial response; SD, Stable disease.

### 3.3 Relevant prognostic value of primary tumor sites

**FIGURE 2:**
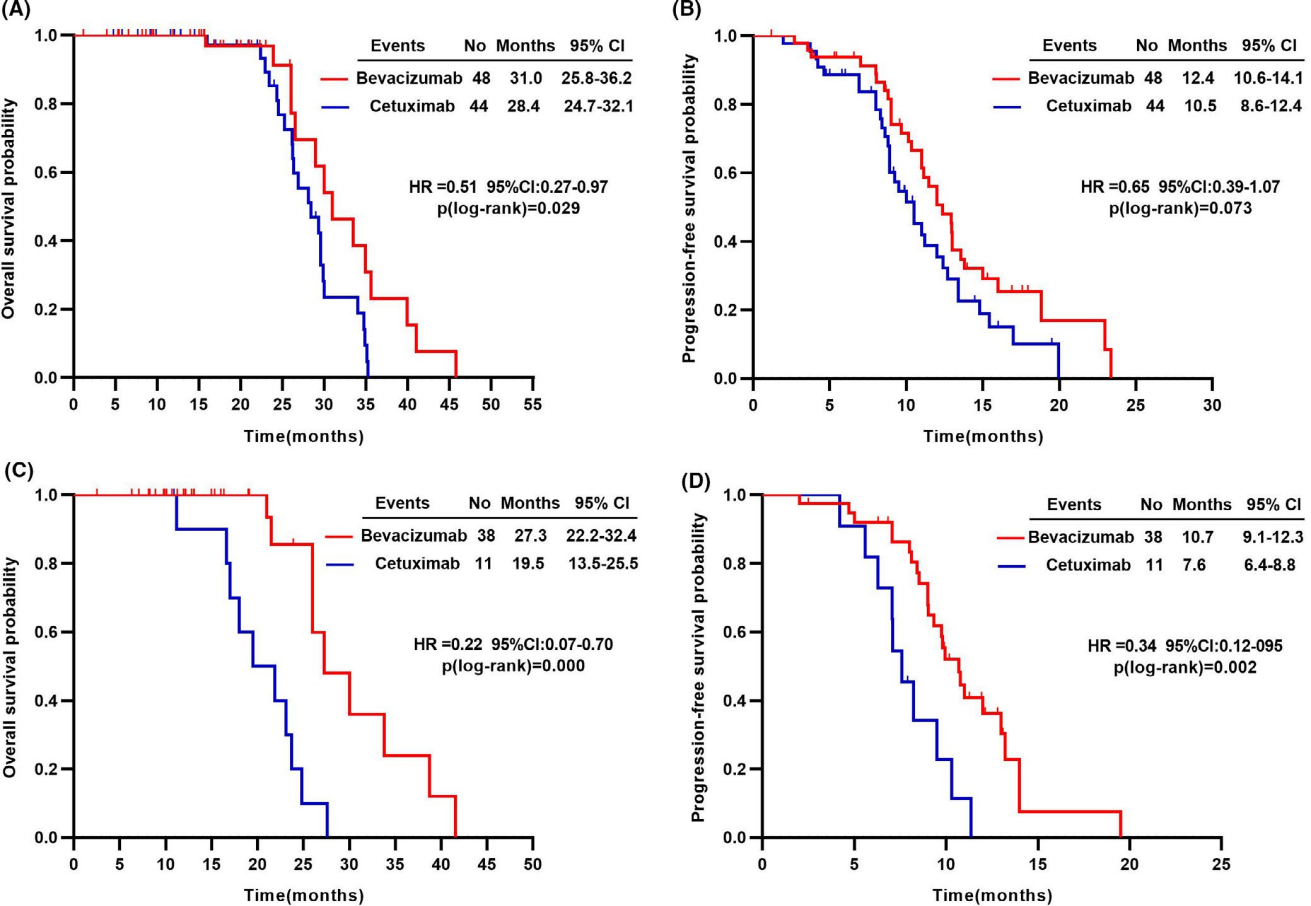
Survival times according to tumor sites in patients with mucinous adenocarcinoma or mucinous component. Kaplan–Meier estimates of overall survival for (A) left‐side (C) right‐side colorectal cancer patients; progression‐free survival for (B) left‐side (D) right‐side colorectal cancer patients

Among patients with MA/MC, median OS in bevacizumab group was significantly better than that in cetuximab group, regardless of the tumor locations (Figure 2A and C). For left‐side colorectal patients, median PFS and ORR was numerically in favor of the bevacizumab group (12.4 vs. 10.5 months, crude HR = 0.65, *p* = 0.073; 56.3 vs. 54.5%, *p* = 0.869) (Figure 2B; Table 4). For right‐side colorectal patients, there were significant difference of median OS and PFS between the bevacizumab and the cetuximab groups (27.3 vs. 19.5 months, crude HR = 0.22, *p* < 0.001; 10.7 vs. 7.6 months, crude HR = 0.34, *p* = 0.002); (Figure 2C and D). ORR of bevacizumab group was numerically higher than that of cetuximab group (50.0% vs. 45.5%, *p* = 0.791) (Table 4).

**FIGURE 3:**
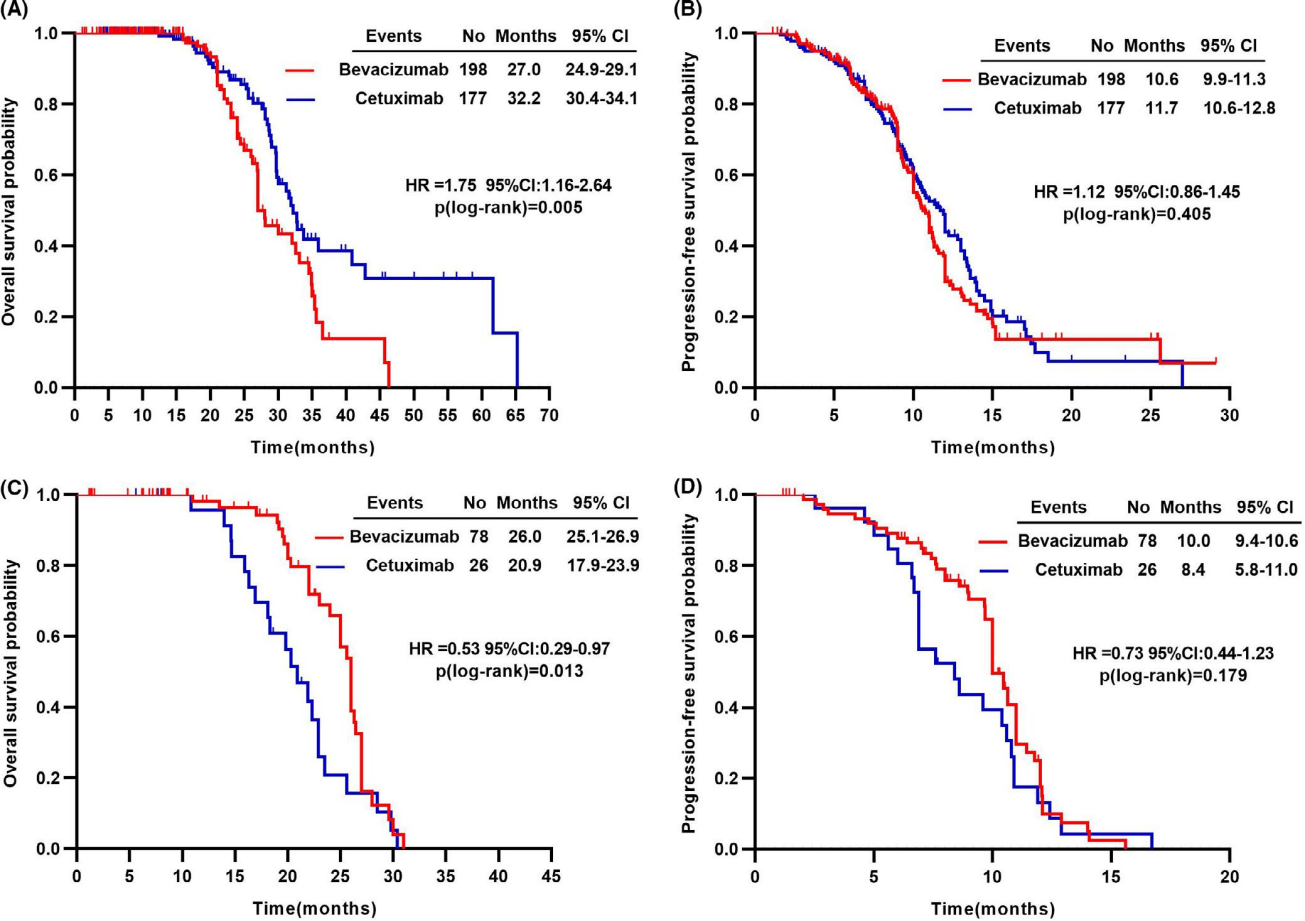
Survival times according to tumor sites in patients with non‐mucinous adenocarcinoma. Kaplan–Meier estimates of overall survival for (A) left‐side (C) right‐side colorectal cancer patients; progression‐free survival for (B) left‐side (D) right‐side colorectal cancer patients

By contrast, among patients with NMA, median OS and ORR for left‐side colorectal patients treated with bevacizumab‐based therapy were worse than those of patients treated with cetuximab‐based therapy (27.0 vs. 32.2 months, crude HR = 1.75, *p* = 0.005; 54.0 vs. 69.5%, *p* = 0.002) (Figure 3A; Table 4). No significant difference for PFS was observed between these two groups (Figure 3B). Nevertheless, for right‐side colorectal patients, both median OS and PFS favored the bevacizumab‐based therapy (26.0 vs. 20.9 months, crude HR = 0.53, *p* = 0.013; 10.0 vs. 8.4 months, crude HR = 0.73, *p* = 0.179) (Figure 3C and D). ORR was comparable between the bevacizumab‐based and cetuximab‐based treatment groups (Table 4). The trends of OS and PFS, either in patients with MA/MC or in patients with NMA, were still hold after adjusting for confounding factors (Table S1).

### 3.4 Relevant prognostic value of combined chemotherapy regimens

**FIGURE 4:**
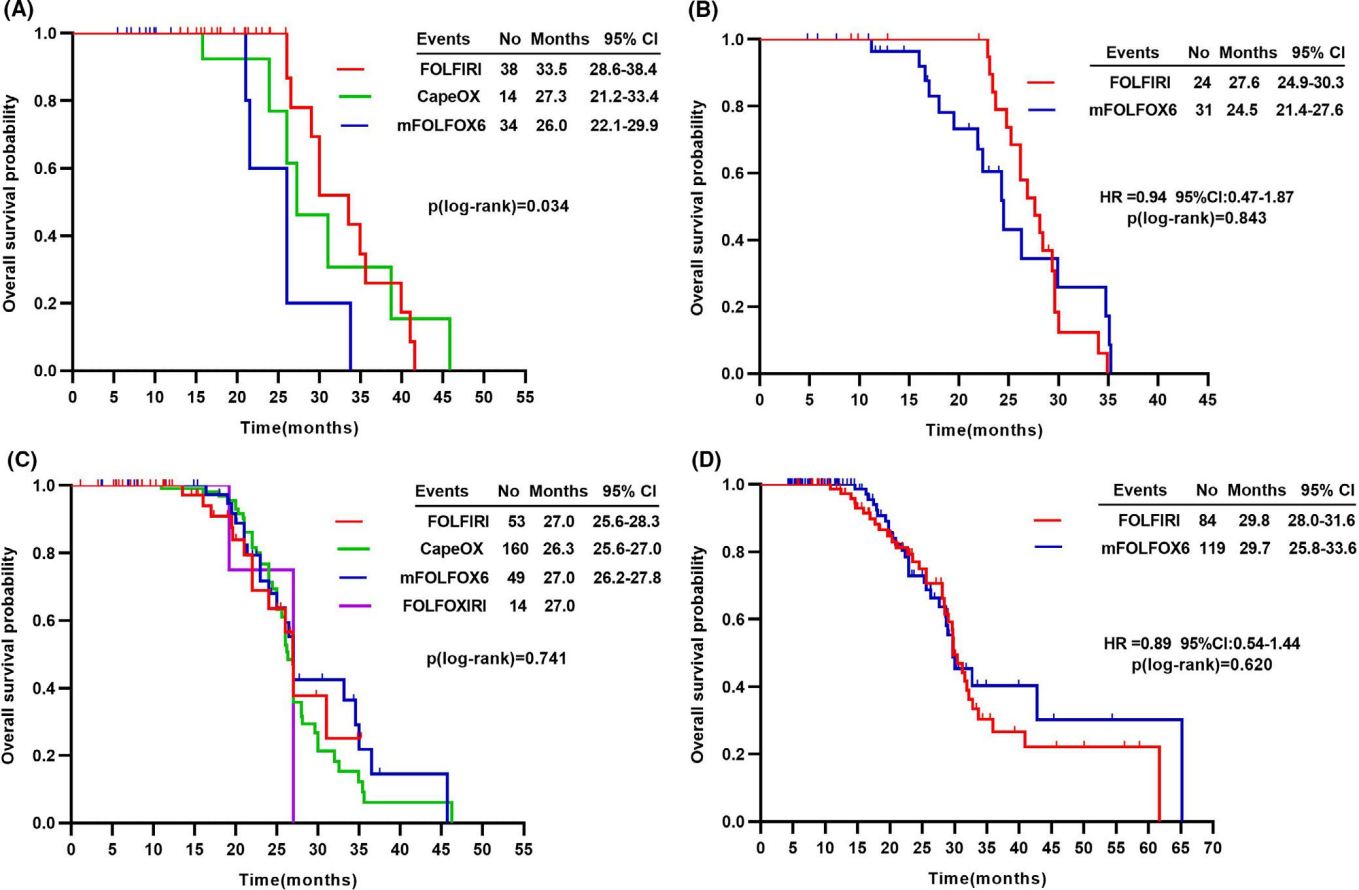
Survival times according to combined chemotherapy regimens. Abbreviations: CapeOx, capecitabine, oxaliplatin; FOLFIRI, folinic acid, fluorouracil, and irinotecan; FOLFOX, folinic acid, fluorouracil, and oxaliplatin; FOLFOXIRI, folinic acid, fluorouracil, irinotecan, and oxaliplatin; MA, mucinous adenocarcinoma; MC, mucinous component; NMA, non‐mucinous adenocarcinoma. Kaplan‐Meier estimates of overall survival for MA/MC patients treated with (A) bevacizumab‐based (B) cetuximab‐based therapy; for NMA patients treated with (C) bevacizumab‐based (D) cetuximab‐based therapy

When considering the different combined‐regimens of chemotherapy, for MA/MC patients treated with bevacizumab, median OS was markedly longer in combination to FOLFIRI than oxaliplatin (OXA)‐based regimens; (Figure 4A). Similarly, median OS was numerically favored the FOLFIRI regimens plus cetuximab in MA/MC patients (Figure 4B). However, in NMA patients treated with either bevacizumab or cetuximab, median OS was comparable among different combined‐chemotherapy regimens (Figure 4C and D).

### 3.5 Subgroup analysis

**FIGURE 5:**
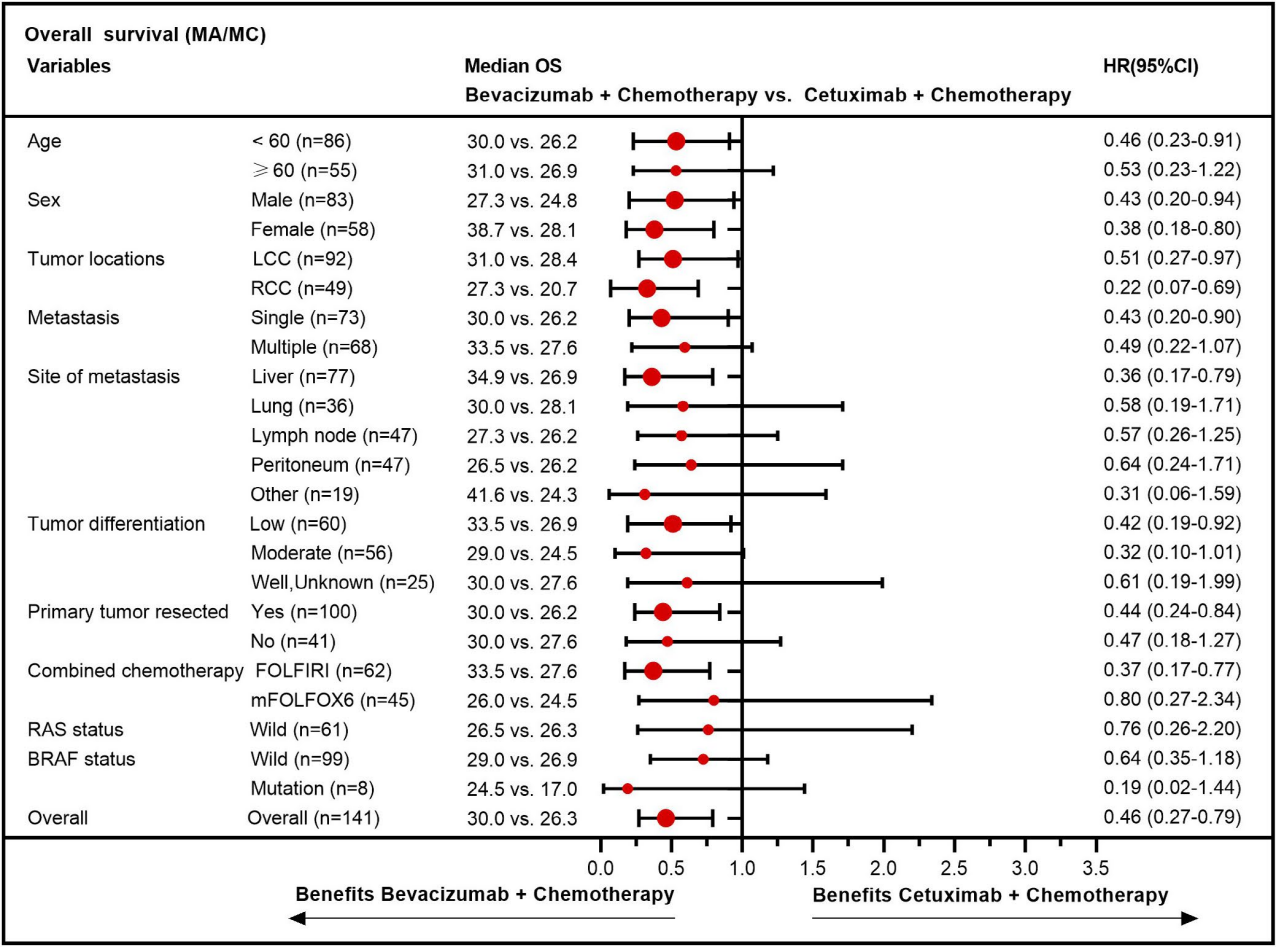
Forest plot of demographic‐ and biomarker‐defined subgroup analyses regarding the overall survival in patients with MA/MC. Abbreviations: FOLFIRI, folinic acid, fluorouracil, and irinotecan; FOLFOX, folinic acid, fluorouracil, and oxaliplatin; LCC, left‐side colorectal cancer; MA, mucinous adenocarcinoma; MC, mucinous component; OS, overall survival; RCC, right‐side colorectal cancer

**FIGURE 6:**
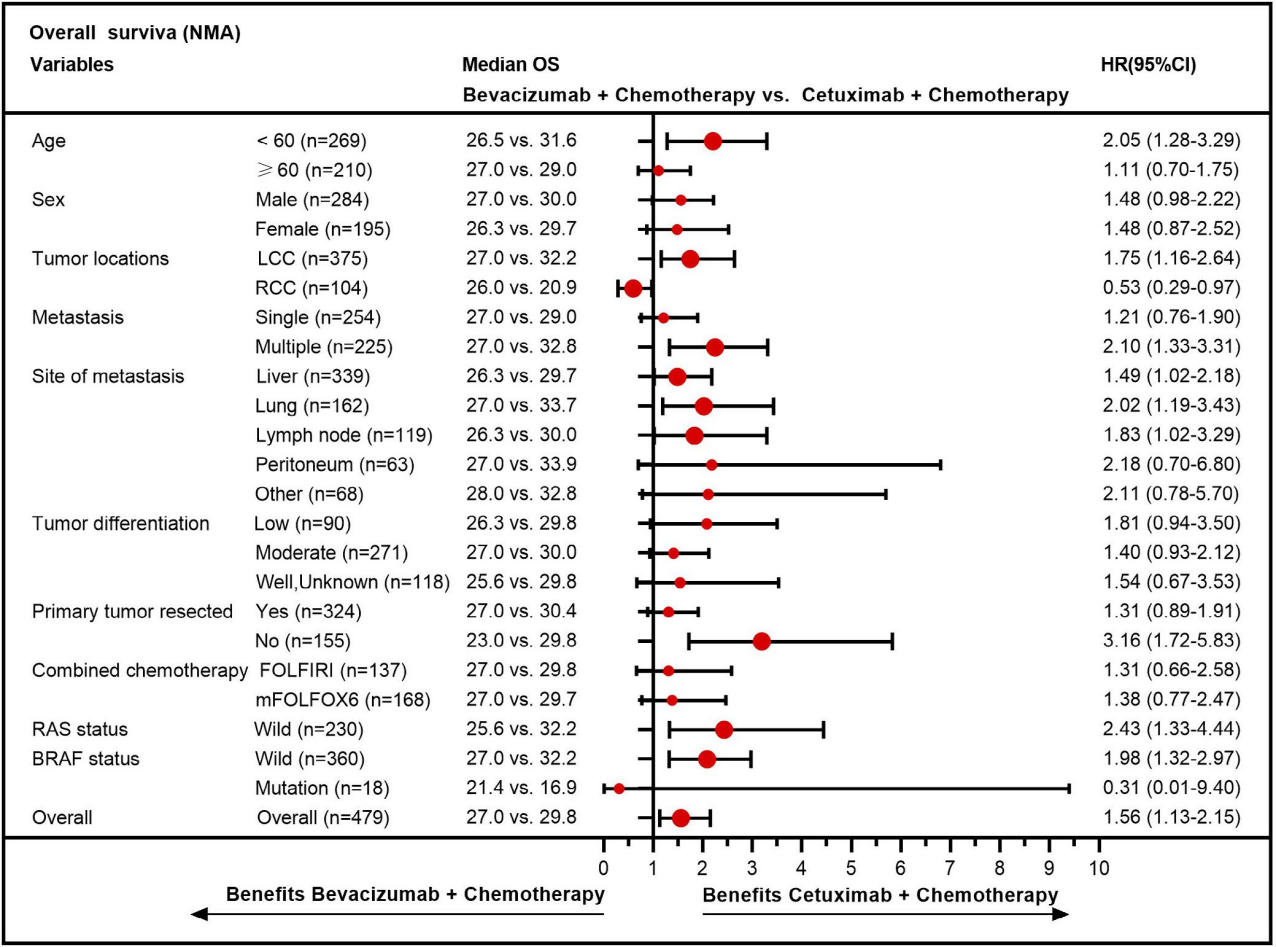
Forest plot of demographic‐ and biomarker‐defined subgroup analyses regarding the overall survival in patients with NMA. Abbreviations: FOLFIRI, folinic acid, fluorouracil, and irinotecan; FOLFOX, folinic acid, fluorouracil, and oxaliplatin; LCC, left‐side colorectal cancer; NMA, non‐mucinous adenocarcinoma; OS, overall survival; RCC, right‐side colorectal cancer

As shown in Figure 5, in patients with MA/MC, the efficacy of bevacizumab‐based chemotherapy was higher in nearly all subgroups. Among NMA patients, more survival beneficial factors could be obtained in the cetuximab group than those in the bevacizumab group, except for the location of right‐side colorectal and BRAF‐mutant status (Figure 6). The results for NMA patients with BRAF‐mutant may be subject to an imbalance baseline characteristic and small patient number in this group. Notably, treatment activity was independent of RAS and BRAF status for mCRC patients with MA/MC, while cetuximab‐based chemotherapy was preferred to recommend for RAS or BRAF wild patients in NMA population.

## 4 DISCUSSION

The optimal systemic treatment of mCRC patients with MA/MC remains to be determined. To the best of our knowledge, the present study is the first retrospective analysis to investigate the efficacy of bevacizumab or cetuximab combined with various chemotherapy regimens in mCRC patients with MA/MC. Our findings suggested that mCRC patients with MA/MC benefited more from the first‐line bevacizumab‐based therapy, regardless of the tumor location. This was highly consistent in nearly all subgroups. For patients with NMA, cetuximab‐based chemotherapy is considered the best option for patients with left‐side mCRC only. When it comes to combined regimens, FOLFIRI plus bevacizumab may be the optimal regimen for patients with MA/MC.

Our results were slightly different from a previous study by You et al.^28^ They reported that primary tumor locations could predict the clinical prognosis of bevacizumab‐treated patients with RAS/BRAF‐wild mCRC and that bevacizumab combined with FOLFIRI regimen was more suitable for patients with left‐sided mCRC. When pathological classification was included, we found that patients with mCRC with MA/MC, either left‐ or right‐side mCRC could benefit from bevacizumab‐based therapy. In another study,^29^ You et al. demonstrated that bevacizumab‐based treatment is an optimal first‐line therapy for right‐sided RAS‐wild mCRC, which is consistent with the outcome of our study that bevacizumab plus chemotherapy is the best regimen for patients with right‐side mCRC, with either MA/MC or NMA.

The genetic/molecular characteristics of CRC may help in explaining these consequences: CMSs offer a comprehensive understanding of the essential characteristics of CRC,^10^ and its classification has been reported as a predictive prognostic marker for mCRC survival.^26^ The highest proportion of CMS1 and the lowest rate of CMS2 were found in CRC patients with MA/MC.^25^ Large clinical trials confirmed that patients with CMS1 cancers achieved more benefit from bevacizumab‐based than cetuximab‐based chemotherapy.^26^ CMS1 was found to be highly correlated with microenvironmental signatures,^30^ such as T‐lymphocyte infiltration triggered vessel normalization,^31^, ^32^ and M1/2 polarization of inflammatory tumor‐associated macrophages,^31^ which may be affected by the action of bevacizumab. However, patients with CMS2 had significantly better survival when chemotherapy was administered in combination with cetuximab compared with that when administered in combination with bevacizumab.^26^ CMS2 cancers, which rarely exist in patients with MA/MC and is characterized by the activation of the EGFR pathway may be associated with enhanced sensitivity to cetuximab.^33^, ^34^ Moreover, MA/MC is associated with high‐frequency of RAS and BRAF mutations as well as alterations in MAPK signaling pathways,^9^ which could result in activation of the EGFR pathway. The CpG island methylator phenotype, an epigenetic mechanism of gene silencing, is commonly observed in MA/MC neoplasms^35^ and EGFR promoter methylation may lead to inefficacy of the anti‐EGFR monoclonal antibodys.^36^

In terms of epidemiological features of mCRC patients with MA/MC, the incidence (141/620, 22.7%) in our study was greater than that previously reported in Asia (5%–10%).^37^ Considering that the clinicopathological characteristic of MC resembled that of MA in the mCRC population,^19^, ^38^ we incorporated patients with MC and MA into one group. Other characteristics of MA/MC in our study, such as higher rates of right‐side (34.8%), poor differentiation (42.6%), RAS‐mutant (43.5%), BRAF‐mutant (7.5%), MSI (5.0%), and the presence of peritoneal metastasis (33.3%), were similar to those reported in previous studies.^39^, ^40^

The results presented here should be considered as preliminary and should be verified in large prospective studies. Only two retrospective studies that investigated whether mCRC patients with MA/MC can benefit from anti‐VEGF or anti‐EGFR treatment have been published recently. Similar to our study, Moretto et al.^41^ also found that mCRC patients with MA/MC did not benefit from anti‐EGFR treatment, irrespective of sidedness. In their study, only 22 patients with MA/MC (11 left‐ and 11 right‐sided tumors) receiving an unknown line of therapy (cetuximab, panitumumab, cetuximab plus chemotherapy) was included, and a median OS of 6.5 months for MA/MC vs. 16.7 months for NMA was obtained. In contrast, our study included 55 patients with MA/MC (44 left‐ and 11 right‐sided tumors) who received first‐line cetuximab combined chemotherapy, and achieved a relatively longer median OS (26.3 months for MA/MC and 29.8 months for NMA). Discrepancies between the present reports and previous findings may be attributable to analytical limitations imposed by relatively small patient numbers, differences in the treatment options and the line of therapy assessed.

Another multicenter retrospective analysis performed by Catalano et al.^42^ reported that numerical OS advantage was obtained in MA/MC patients treated with bevacizumab plus chemotherapy. In this study, 94 patients with MA/MC (53 left‐ and 41 right‐sided tumors), who received first‐line bevacizumab plus chemotherapy, had a slight numerical advantage in OS (28.2 months) over those with NMA (27.7 months). Similarly,86 patients with MA/MC (48 left‐ and 38 right‐sided tumors) were included in our study, and a median OS of 30.0 months for MA/MC and 27.0 months for NMA was achieved. Interestingly, similar to the study of Catalano et al., a significantly different OS was obtained between patients with MA/MC treated with FOLFIRI and mFOLFOX6 regimens in the bevacizumab groups. In the cetuximab groups, longer OS was also observed for patients with MA/MC treated with FOLFIRI compared to those treated with mFOLFOX6. These results seem to corroborate those of other research: Longer OS was found in mCRC patients who received FOLFIRI than in those who received FOLFOX when the tumor classifier was imbued with Wnt signaling activation,^43^ in which the characteristics was related to the initiation of mucinous colorectal carcinomas.^44^ From a pharmacological perspective, the overexpression of thymidylate synthase and glutathione S‐transferase‐pi (GSTP1), a marker that is resistant to oxaliplatin as well as 5‐FU^45^ and the downregulation of UGT1A enzymes, a sensitive ingredient to irinotecan,^46^ were demonstrated in MA/MC tumors.

Although improved OS and PFS of MA/MC was achieved in the bevacizumab group regardless of tumor locations, comparable the bevacizumab and cetuximab groups in our study had comparable ORR. However, observably reduced ORR was observed for patients with MA/MC compare with patients with NMA in the cetuximab‐treated group. These results may be attributed to the following reasons: A minimum number of tumor cells of MA/MC could react to systemic therapy, whereas a large amount of mucin could not respond to the treatment. Therefore, the tumor volume did not shrink and lead to false negative results.^5^ Of note, Mekenkamp et al.^40^ confirmed that the disease control rate (CR + PR + SD) was similar between patients with MA/MC and NMA. This may be attributed to RECIST criteria, which may not be the best method to evaluate the tumor response in patients with MA/MC.^5^

In the subgroup analysis, nearly all factors including wild‐RAS status favored bevacizumab‐based chemotherapy for patients with MA/MC. Notably, published large clinical trials have proven that the first‐line therapy of cetuximab‐based treatment was recommended for RAS‐wild left‐side mCRC patients.^21^, ^47^, ^48^ The contradictory result may be attributed to the differences in biological behavior, gene expression, CMS distribution and clinical characteristics between patients with MA/MC and CAC. For patients with NMA, additional factors beneficial to survival, except for the right‐side colorectal cancer and BRAF‐mutant status, favored the cetuximab group over the bevacizumab group. Patients with right‐side mCRC who were insensitive to cetuximab have been reported in a large number of research studies.^24^, ^49^, ^50^ In addition, some have also found that mCRC patients with BRAF‐mutant are more likely to benefit from bevacizumab‐based therapy than cetuximab‐based therapy.

Despite the rigorous design of the study, several limitations of this study should be acknowledged: Given the single‐center retrospective nature of the study, the limited number of samples and biases related to imbalances in baseline characteristics between the treatment arms could not be circumvented. Moreover, a powerful stratification of MA/MC affected by chemotherapy was not possible. The interactions between targeted and cytotoxic agents may have an impact on the predictive effect of MA/MC classification. Finally, eligible patients were enrolled in September, 2013, when first‐line cetuximab in combination with chemotherapy was made available to mCRC patients regardless of tumor sites according to NCCN guidelines. As a result, a small number of patients with right‐side mCRC (who were later proven to be insensitive to cetuximab) were incorporated into the cetuximab groups.

In conclusion, our findings suggest that patient stratification based on conventional pathological classification (e.g. MA/MC) should be considered in tailoring the individualized optimal treatment for mCRC. First‐line bevacizumab‐based chemotherapy is the preferred treatment for patients with MA/MC, regardless of tumor location. Among all combined chemotherapy regimens, FOLFIRI may be the optimal candidate. For patients with NMA, cetuximab‐based chemotherapy is considered as the best option for patients with left‐side mCRC only. Therefore, this retrospective study may provide evidence for clinical practice regarding the selection of chemotherapy in combination with cetuximab or bevacizumab for mCRC patients with MA/MC. Accordingly, further clarity may be achieved from the analysis of future trials of prospective and already published trials of retrospective studies.

## CONFLICT OF INTEREST

None declared.

## ETHICS APPROVAL

The protocol was approved by the Ethics Committee of West China Hospital.

## Supporting information


Table S1

Table S2

## Data Availability

The data that support the findings of this study are available from the corresponding author upon reasonable request.
