# Supplementary material for: First‐line therapy of bevacizumab plus chemotherapy versus cetuximab plus chemotherapy for metastatic colorectal cancer patients with mucinous adenocarcinoma or mucinous component
Source: Cancer Med. 2021 May 3;10(10):3388–402. doi: 10.1002/cam4.3876 (PMC8124114; doi:10.1002/cam4.3876)
Supplement: Supplementary file 1 — Table S1 [file CAM4-10-3388-s002.docx]

**Supplementary Table 1.** Multivariate analyses of primary tumor location on efficacy in the colorectal cancer patients with MA/MC or with NMA.

| **MA/MC** | **Bev vs. Cet** |  | **Bev vs. Cet** |  | **Bev vs. Cet** |  |
| --- | --- | --- | --- | --- | --- | --- |
|  | **Total (n=141)** |  | **Left colorectal (n=92)** |  | **Right colorectal (n=49)** |  |
|  | Adjusted HR^*^ (95%CI) | *p* value | Adjusted HR^*^ (95%CI) | *p* value | Adjusted HR^*^ (95%CI) | *p* value |
| OS | 0.24 (0.12-0.50) | **<0.001** | 0.18 (0.06-0.58) | **0.004** | 0.03 (0.00-0.23) | **0.001** |
| PFS | 0.61 (0.39-0.96) | **0.033** | 0.63 (0.33-1.19) | 0.150 | 0.27 (0.11-0.64) | **0.003** |
| **NMA** | **Total (n=479)** |  | **Left colorectal (N=375)** |  | **Right colorectal (n=104)** |  |
|  | Adjusted HR^*^ (95%CI) | *p* value | Adjusted HR^*^ (95%CI) | *p* value | Adjusted HR^*^ (95%CI) | *p* value |
| OS | 1.57 (1.10-2.25) | **0.013** | 1.87 (1.18-2.96) | **0.007** | 0.47 (0.26-0.87) | **0.015** |
| PFS | 1.12 (0.88-1.43) | 0.342 | 1.15 (0.87-1.52) | 0.328 | 0.74 (0.45-1.23) | 0.250 |

**Abbreviations MA:** mucinous adenocarcinoma; **MC:** mucinous component; **NMA:** non-mucinous adenocarcinoma; **OS:** overall survival; **PFS:** progression-free survival; **Bev:** bevacizumab; **Cet:** cetuximab.

**^*^** After adjusting for potential covariates: sex, age, tumor differentiation, tumor metastatic status, primary tumor resection
