# Supplementary material for: First‐line therapy of bevacizumab plus chemotherapy versus cetuximab plus chemotherapy for metastatic colorectal cancer patients with mucinous adenocarcinoma or mucinous component
Source: Cancer Med. 2021 May 3;10(10):3388–402. doi: 10.1002/cam4.3876 (PMC8124114; doi:10.1002/cam4.3876)
Supplement: Supplementary file 2 — Table S2 [file CAM4-10-3388-s001.docx]

**Supplementary Table 2.** RAS/BRAF/MMR status in the left and right colon.

| **Gene Status** | **Total** | **Left colon** | **Right colon** |
| --- | --- | --- | --- |
|  | 620 (100%) | 467 (75.3%) | 153 (24.7%) |
| ***RAS* status** |  |  |  |
| Wild | 291 (46.9) | 239 (51.2) | 52 (34.0) |
| Mutant | 195 (31.5) | 144 (30.8) | 51 (33.3) |
| Unknown | 134 (21.6) | 84 (18.0) | 50 (32.7) |
| ***BRAF* status** |  |  |  |
| Wild | 459 (74.0) | 363 (77.7) | 96 (62.7) |
| Mutant | 26 (4.2) | 19 (4.1) | 7 (4.6) |
| Unknown | 135 (21.8) | 85 (18.2) | 50 (32.7) |
| ***MMR* status** |  |  |  |
| MSS | 331 (53.4) | 261 (55.9) | 70 (45.8) |
| MSI | 8 (1.3) | 5 (1.1) | 3 (2.0) |
| Unknown | 281 (45.3) | 201 (43.0) | 80 (52.3) |

**Abbreviations MMR** Mismatch repair; **MSS** Microsatellite stable; **MSI** Microsatellite instability.
